# Supplementary material for: Risk assessment of pesticide residues ingestion in food offered by institutional restaurant menus
Source: PLoS One. 2024 Dec 18;19(12):e0313836. doi: 10.1371/journal.pone.0313836 (PMC11654986; doi:10.1371/journal.pone.0313836)
Supplement: S1 Table — (DOCX) [file pone.0313836.s001.docx]

**S1. Supplementary material**

| Active ingredient | Frequency of foods on the menu | Toxicological classification* | Agronomic classification ** | Log P | Classification of bioaccumulation potential |
| --- | --- | --- | --- | --- | --- |
| Abamectin | 19x | Class IV - Slightly toxic product | Acaricide, insecticide and nematicide | 3.8 | High*** |
| Acephate | 16x | Class IV - Slightly toxic product | Insecticide and acaricide | -0.8 | Low*** |
| Acetamiprid | 23x | Class IV - Slightly toxic product | Fungicide | 1.4 | Low*** |
| Acibenzolar-s-methyl | 17x | Class V - Product unlikely to cause acute harm | Fungicide | 3.1 | High*** |
| Alpha-cypermethrin | 22x | Class V - Product unlikely to cause acute harm | Fungicide | 6.0 | High*** |
| Azoxystrobin | 35x | Class IV - Slightly toxic product | Systemic fungicide | 3.7 | High*** |
| Beta-cypermethrin | 18x | Class IV - Slightly toxic product | Insecticide | 6.9 | High*** |
| Bifenthrin | 25x | Class IV - Slightly toxic product | Insecticide, anticide and acaricide | 6 | High*** |
| Boscalida | 19x | Class V - Product unlikely to cause acute harm | Fungicide | 2.96 | Moderate*** |
| Buprofezin | 18x | Class V - Product unlikely to cause acute harm | Insecticide and acaricide | 3.8 | High*** |
| Carbaryl | 19x | Class IV - Slightly toxic product | Insecticide | 2.4 | Low*** |
| Carbendazim | 12x | Class V - Product unlikely to cause acute harm | Fungicide | 1.52 | Low*** |
| Kasugamycin | 24x | Class III - Moderately toxic product | Fungicide and bactericide | -1.9 | Low*** |
| Cyantraniliprole | 25x | Unrated product | Insecticide | 3.9 | High*** |
| Cymoxanil | 17x | Class V - Product unlikely to cause acute harm | Fungicide | 0.7 | Low*** |
| Cypermethrin (all isomers) | 26x | Class IV - Slightly toxic product | Insecticide | 6.60 | High*** |
| Cyproconazole | 14x | Class III - Moderately toxic product | Insecticide | 2.90 | Moderate*** |
| Ciprodinil | 17x | Class V - Product unlikely to cause acute harm | Fungicide | 3.1 | High*** |
| Clethodim | 30x | Class V - Product unlikely to cause acute harm | Herbicide | 3.8 | High*** |
| Chlorantraniliprole | 10x | Class IV - Slightly toxic product | Systemic Insecticide | 2.9 | Moderate*** |
| Chlorfenapyr | 22x | Class V - Product unlikely to cause acute harm | Insecticide and acaricide | 4.5 | High*** |
| Chlorothalonil | 35x | Class III - Moderately toxic product | Contact fungicide | 2.9 | Moderate*** |
| Clotianidin | 17x | Class IV - Slightly toxic product | Insecticide | 0.7 | Low*** |
| Cresodim-methyl | 12x | Unrated product | Contact fungicide | 3.40 | High*** |
| Deltamethrin | 30x | Class IV - Slightly toxic product | Insecticide and anticide | 6.2 | High*** |
| Paraquat dichloride | 13x | Class I - Extremely toxic product | Herbicide | -4.5 | Low*** |
| Difenoconazole | 30x | Class V - Product unlikely to cause acute harm | Fungicide | 4 | High*** |
| Dithiocarbamates | 26x | Class III - Moderately toxic product | Herbicide | 0.3 | Low*** |
| Spinetoram | 25x | Class V - Product unlikely to cause acute harm | Insecticide | 5.9 | High*** |
| Spiromesifene | 31x | Class III - Moderately toxic product | Insecticide and acaricide | 5.1 | High*** |
| Etofenproxi | 27x | Class V - Product unlikely to cause acute harm | Insecticide | 7 | High*** |
| Famoxadone | 24x | Class IV - Slightly toxic product | Fungicide | 5 | High*** |
| Fenamidone | 18x | Class III - Moderately toxic product | Fungicide | 4.1 | High*** |
| Fenpyroximate | 14x | Class II - Highly toxic product | Acaricide | 4.9 | High*** |
| Fenpropathrin | 20x | Class III - Moderately toxic product | Insecticide and acaricide | 5.7 | High*** |
| Fluazinam | 16x | Class V - Product unlikely to cause acute harm | Fungicide and acaricide | 6 | High*** |
| Flubendiamide | 14x | Unrated product | Insecticide | 4.2 | High*** |
| Fludioxonil | 21x | Class III - Moderately toxic product | Fungicide | 2.6 | Low*** |
| Fluensulfone | 21x | Class V - Product unlikely to cause acute harm | Nematicide | 2.5 | Low*** |
| Flumioxazine | 15x | Class V - Product unlikely to cause acute harm | Herbicide | 2.55 | Low*** |
| Flupyradifurone | 25x | Class IV - Slightly toxic product | Insecticide | 2.3 | Low*** |
| Flutriafol | 37x | Class III - Moderately toxic product | Systemic fungicide | 2.3 | Low*** |
| Fluxapyroxad | 35x | Class IV - Slightly toxic product | Fungicide | 3.7 | High*** |
| Glyphosate | 23x | Class V - Product unlikely to cause acute harm | Herbicide | -6.28 | Low**** |
| Glufosinate | 12x | Class IV - Slightly toxic product | Herbicide and growth regulator | -0.1 | Low*** |
| Imidacloprid | 27x | Class IV - Slightly toxic product | Insecticide | 1.2 | Low*** |
| Indoxacarb | 30x | Class IV - Slightly toxic product | Insecticide | 4.8 | High*** |
| Isoxaflutole | 11x | Class V - Product unlikely to cause acute harm | Selective systemic herbicide | 2.32 | Low*** |
| Lambda-cyhalothrin | 25x | Class V - Product unlikely to cause acute harm | Insecticide | 7 | High*** |
| Mancozeb | 20x | Class V - Product unlikely to cause acute harm | Fungicide and acaricide | 2.3 | Low**** |
| Mandipropamide | 25x | Class II - Highly toxic product | Insecticide | 3.9 | High*** |
| Metalaxyl-M | 15x | Class III - Moderately toxic product | Fungicide | 1.6 | Low*** |
| Metconazole | 13x | Class I - Extremely toxic product | Fungicide | 3.7 | High*** |
| Methomyl | 22x | Class II - Highly toxic product | Insecticide and acaricide | 0.6 | Low*** |
| Paraquat | 16x | Class I - Extremely toxic product | Herbicide | -4.2 | Low*** |
| Pendimentalin | 12x | Class V - Product unlikely to cause acute harm | Herbicide | 5.20 | High*** |
| Permethrin | 13x | Class IV - Slightly toxic product | Insecticide and anticide | 6.5 | High*** |
| Picoxystrobin | 16x | Class III - Moderately toxic product | Fungicide | 3.6 | High*** |
| Pyraclostrobin | 35x | Class IV - Slightly toxic product | Fungicide | 4.1 | High*** |
| Pyrimethanil | 18x | Unrated product | Fungicide | 2.9 | Moderate*** |
| Pirimicarb | 14x | Class II - Highly toxic product | Insecticide | 1.7 | Low*** |
| Pyriproxyfen | 19x | Class V - Product unlikely to cause acute harm | Insecticide | 4.8 | High*** |
| Profenofos | 20x | Class IV - Slightly toxic product | Insecticide and acaricide | 4.7 | High*** |
| Propamocarb | 20x | Class V - Product unlikely to cause acute harm | Fungicide | 1.12 | Low*** |
| Propiconazole | 17x | Class IV - Slightly toxic product | Fungicide | 3.5 | High*** |
| Saflufenacil | 14x | Class V - Product unlikely to cause acute harm | Herbicide | 2.1 | Low*** |
| Spinosad | 11x | Class III - Moderately toxic product | Insecticide | 2.8 | Moderate*** |
| Sulfoxaflor | 16x | Unrated product | Insecticide | 2.2 | Low*** |
| Tebuconazole | 40x | Class IV - Slightly toxic product | Systemic fungicide | 3.7 | High*** |
| Tebufenozide | 14x | Class IV - Slightly toxic product | Insecticide | 4.25 | High*** |
| Teflubenzuron | 20x | Unrated product | Insecticide | 4.3 | High**** |
| Tetraconazole | 16x | Class IV - Slightly toxic product | Fungicide | 4.4 | High*** |
| Thiabendazole | 20x | Class V - Product unlikely to cause acute harm | Fungicide | 2.5 | Low*** |
| Thiamethoxam | 30x | Class V - Product unlikely to cause acute harm | Contact systemic insecticide | 1.5 | Low*** |
| Thiophanate-methyl | 18x | Class IV - Slightly toxic product | Fungicide and acaricide | 2.6 | Low*** |
| Trifloxystrobin | 37x | Class IV - Slightly toxic product | Fungicide | 4.5 | High*** |
| Trifluralin | 15x | Class V - Product unlikely to cause acute harm | Herbicide | 5.34 | High*** |

*ANVISA^125^ **MAPA^126^ ***PubChem^127^ ****PPDB^128^; Log P: partition coefficient.

*I: extremely toxic; II: highly toxic; III: moderately toxic; IV: slightly toxic; V: unlikely to cause acute harm.
